# Dehydrin CaDHN2 Enhances Drought Tolerance by Affecting Ascorbic Acid Synthesis under Drought in Peppers

**DOI:** 10.3390/plants12223895

**Published:** 2023-11-18

**Authors:** Xin Li, Hao Feng, Sha Liu, Junjun Cui, Jiannan Liu, Mingyu Shi, Jielong Zhao, Lihu Wang

**Affiliations:** 1 School of Landscape and Ecological Engineering, Hebei University of Engineering, Handan 056038, China; xinkebobo@163.com (X.L.); liushassl@163.com (S.L.); cuijunjun@hebeu.edu.cn (J.C.); ljn_19881029@163.com (J.L.); 2 Beijing Key Laboratory of Agricultural Genetic Resources and Biotechnology, Institute of Biotechnology, Beijing Academy of Agriculture and Forestry Sciences, Beijing 100097, China; fenghao@babrc.ac.cn (H.F.); mingyu_shi99@163.com (M.S.); m19963118384@163.com (J.Z.)

**Keywords:** CaDHN2, drought tolerance, ascorbic acid, pepper

## Abstract

Peppers (*Capsicum annuum* L.), as a horticultural crop with one of the highest ascorbic acid contents, are negatively affected by detrimental environmental conditions both in terms of quality and productivity. In peppers, the high level of ascorbic acid is not only a nutrient substance but also plays a role in environmental stress, i.e., drought stress. When suffering from drought stress, plants accumulate dehydrins, which play important roles in the stress response. Here, we isolated an SK_3_-type DHN gene CaDHN2 from peppers. CaDHN2 was located in the nucleus, cytoplasm, and cell membrane. In CaDHN2-silenced peppers, which are generated by virus-induced gene silencing (VIGS), the survival rate is much lower, the electrolytic leakage is higher, and the accumulation of reactive oxygen species (ROS) is greater when compared with the control under drought stress. Moreover, when CaDHN2 (CaDHN2-OE) is overexpressed in *Arabidopsis*, theoverexpressing plants show enhanced drought tolerance, increased antioxidant enzyme activities, and lower ROS content. Based on yeast two-hybrid (Y2H), GST-pull down, and bimolecular fluorescence complementation (BiFC) results, we found that CaDHN2 interacts with CaGGP1, the key enzyme in ascorbic acid (AsA) synthesis, in the cytoplasm. Accordingly, the level of ascorbic acid is highly reduced in CaDHN2-silenced peppers, indicating that CaDHN2 interacts with CaGGP1 to affect the synthesis of ascorbic acid under drought stress, thus improving the drought tolerance of peppers. Our research provides a basis for further study of the function of DHN genes.

## 1. Introduction

Drought stress is widely recognized as the predominant constraint on global plant growth and crop yield, causing adverse outcomes including the inhibition of vital enzymatic processes, perturbation of cellular membrane integrity, diminished nutrient provision, and the excessive generation of reactive oxygen species (ROS) [1,2]. To survive adverse environmental conditions, plants have evolved different response strategies. At the physiological level, stomata close to reduce water loss [3], more lateral roots emerge to extend water uptake, and the photosynthesis rate reduces to minimize water use [4]. At the molecular level, stress-related genes are induced to maintain cell homeostasis, including transcription factors, kinase, protease, dehydrins, etc. [5,6,7], which have been identified in many species of rice, tomatoes, and peppers. Exploring the capacity of plants to withstand stress and their ability to adapt to challenging environmental conditions can provide valuable insights into addressing environmental issues.

The pepper *Capsicum annuum* L. is a very important horticultural crop, which is very popular and cultivated worldwide because of its nutrient value. Peppers are one of the most ascorbic acid (also called vitamin C)-enriched vegetables and are called “the King of Vitamin C in vegetable” in China. Like other plants, peppers also suffer from abiotic stresses, including drought, chilling, and salt conditions. Under these detrimental conditions, pepper growth is highly inhibited, and pepper death is also accelerated. Many stress-induced genes have been identified that affect the pepper drought response, such as E3 Ubiquitin Ligase [8,9,10,11], CIPK3 [12], heat-shock proteins (Hsp) [13], serine–threonine kinase [14], transcriptional factors [15,16], dehydrins [17], and other abiotic stress-induced proteins.

Ascorbic acid (AsA), commonly known as vitamin C, possesses antioxidant attributes and plays a pivotal role in crucial metabolic pathways within plants. These pathways include photosynthesis, photoprotection, and responses to environmental stressors and pathogenic assaults [18,19]. AsA is commonly recognized for its antioxidative properties and serves as a protective agent against the detrimental impacts of the accumulation of reactive oxygen species (ROS) resulting from oxidative damage induced by environmental stressors [18,20]. Augmenting the endogenous AsA levels with genetic engineering or exogenous application has been demonstrated as an effective strategy to alleviate the deleterious effects of environmental stress on plant physiology [21,22,23]. Notably, the application of AsA has been shown to significantly enhance parameters such as the germination rate, germination percentage, and seedling fresh biomass [20,24,25,26]. Furthermore, AsA has been observed to enhance several physiological aspects of plants, which improves proline levels, relative water content (RWC), leaf anatomical characteristics, and chlorophyll concentration. Additionally, AsA contributes to the elevation of antioxidant enzyme activities, including superoxide dismutase (SOD), catalase (CAT), peroxidase (POD), proline, and soluble carbohydrate contents [27,28,29]. These mechanisms collectively function to reduce the levels of anthocyanins, malondialdehyde (MDA), and hydrogen peroxide (H_2_O_2_).

In other plants, the biosynthesis of ascorbic acid (AsA) occurs through multiple pathways, including the D-mannose/L-galactose pathway, the L-galactose pathway, and pathways associated with cell wall pectins; the myo-inositol.D-mannose/L-galactose pathway is the most well-known primary pathway and has been reported in the leaves and fruits of pepper plants. In this complex process, GDP-L-galactose phosphorylase (GGP) is a rate-limiting enzyme that plays a vital role in AsA synthesis and is a major determinant of AsA concentration [30]. The overexpression of GGP in tomatoes could improve the AsA content and yield [31,32], in turn increasing the activity of antioxidant enzyme activity, such as SOD, CAT, and POD.

Dehydrins are widely distributed in many plant species [33,34] and are D11 or group 2 late-embryogenesis-abundant (LEA) proteins. There are five types of DHNs based on the existence of segments: Y_m_K_n_, Y_m_SK_n_, K_n_, K_n_S, and SK_n_ in plants [35]. The recent identification of the F-segment has expanded the classification of architectural structures to a total of seven, encompassing F_n_K_n_ and F_n_SK_n_ architectures within this categorization [36]. Almost all dehydrins have a K-segment, which can be accurately described as (XKXGXX(D/E)KXK(D/E)KXPG). A K-segment can form amphipathic helices and confers dehydrins with both hydrophilic and hydrophobic characteristics, which play positive roles in protecting lipid membranes and cellular macromolecules. The S-segment is a cluster of serine residues, has other conserved residues, and can be described as (LHR(S/T)GS4–6(S/D/E)(D/E)3) [37]. Normally, the S-segment is a target for phosphorylation. The “Y-segment” contains a consensus amino acid sequence (V/T)DEYGNP in the amino terminus. Moreover, dehydrins are characterized by the absence of cysteine and tryptophan residues while being abundant in polar and charged amino acid residues such as glycine. These distinctive features render dehydrins intrinsically disordered proteins, implying their infrequent adoption of well-defined secondary and tertiary structures. As a result, they exhibit high flexibility and have a tendency to interact easily with various biomolecules within plant cells, including, but not limited to, proteins, nucleotides, ions, and membranes [38]. 

Multiple dehydrin genes have been found in Arabidopsis, wheat, maize, tomatoes, rice, and peppers [39]. The accumulation of dehydrins enhances drought, chilling, and salt tolerance [40,41]. In plant cells, dehydrins can maintain cell stabilization and homeostasis They help prevent cell dehydration, eliminate free radicals, protect macromolecules, and bind metal ions [37,42,43]. Li et al. found that dehydrin MtCAS31, which functions as a cargo receptor, participates in autophagy degradation in response to drought stress in Medicago [40]. Recently, Saruhan et al. found that dehydrin affects ROS homeostasis under osmotic stress [44]. In peppers, CaDHN3 enhances drought tolerance by reducing ROS accumulation [17]. However, the mechanism by which dehydrins affect ROS scavenging has not yet been revealed. Peppers are known for their high content of ascorbic acid (AsA), which not only serves as an indicator of pepper quality but also acts as an antioxidant during times of stress. In our research, we aim to investigate the correlation between drought and pepper quality, with the goal of enhancing the quality of peppers under drought conditions. Maintaining an appropriate level of AsA content under drought can significantly improve both the quality of peppers and their tolerance to drought. Our research provides valuable insights into the role of dehydrins and expands our understanding of how dehydrins control ascorbic acid levels in pepper plants under drought stress.

## 2. Results

### 2.1. CaDHN2 Is a SK_3_-Type Dehydrin 

**Figure 1:**
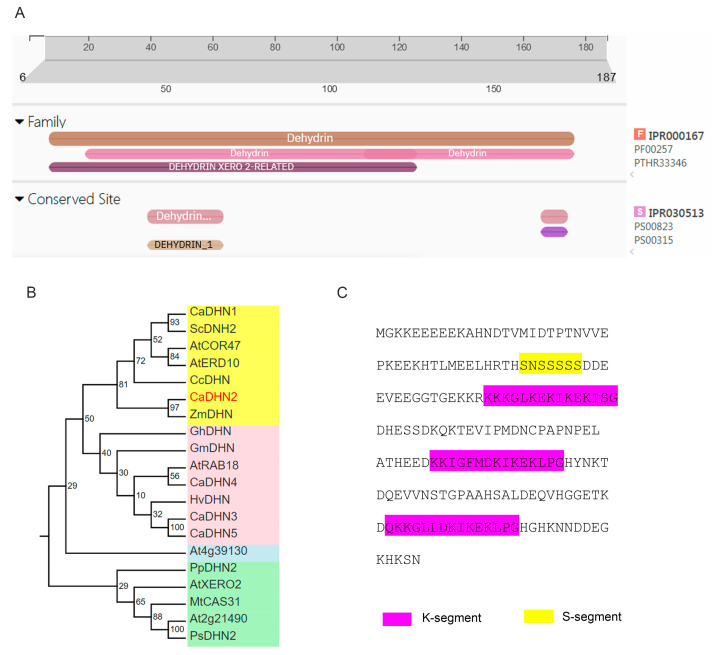
Cluster analysis of CaDHN2. (**A**) Conserved domain analysis of CaDHN2 with Pfam. (**B**) Phylogenetic analysis of dehydrins in different species. The numbers represent the support value. Different colors represent the different subgroup of dehydrins. (**C**) S- and K-segments in CaDHN2.

The full-length CDS of CaDHN2 was successfully cloned from *Capsicum annuum* L. CaDHN2 is a classic dehydrin protein encoding 187 amino acids, with a molecular weight of 17 KDa (Figure 1A). A phylogenetic tree was constructed using different proteins, including DHN subfamilies from other species. CaDHN2 showed homology with DHN proteins and was closest with ZmDHN (Figure 1B). Furthermore, the sequence analysis of CaDHN2 revealed that CaDHN2 contains one S segment and three K segments, indicating that CaDHN2 is an SK_3_ dehydrin belonging to the SnKn-type subfamily (Figure 1C).

### 2.2. CaDHN2 Is Induced by Drought, Salt, and ABA

**Figure 2:**
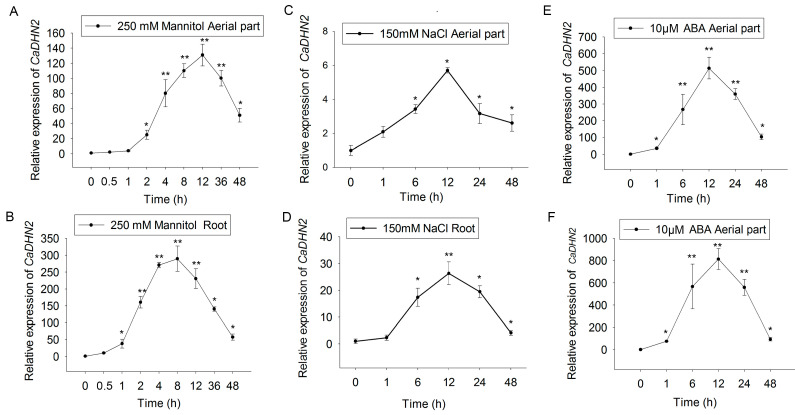
The relative expression of *CaDHN2*. The relative expression of *CaDHN2* was measured with qRT-PCR. (**A**) The relative expression of *CaDHN2* in aerial parts under 250 mM mannitol. (**B**) Relative expression of *CaDHN2* in roots under 250 mM mannitol. (**C**) The relative expression of *CaDHN2* in aerial parts under 150 mM NaCl. (**D**) The relative expression of *CaDHN2* in roots under 150 mM NaCl. (**E**) The relative expression of *CaDHN2* in aerial parts under 10 μM ABA. (**F**) The relative expression of *CaDHN2* in roost under 10 μM ABA. The values were normalized to actin expression. Statistical significance was assessed using Student’s *t*-test, comparing the obtained mean and standard deviation values from three independent experiments to the control. Significance is denoted with asterisks (* *p* < 0.05, ** *p* < 0.01) in the results.

The expression of dehydrins is usually considered a potential marker of abiotic stress [38,45]. For further study, we first examined the relative expression of *CaDHN2* using qRT-PCR. The results showed that the expression of *CaDHN2* was highly induced by drought (simulated with 250 mM mannitol treatment) both in the roots (Figure 2A) and aerial parts (Figure 2B). Similarly, the expression of *CaDHN2* was also induced by salt stress (150 mM NaCl) in both the roots (Figure 2C) and aerial parts (Figure 2D). However, the highest induction levels were observed under drought stress, suggesting that CaDHN2 primarily functions in response to drought. The highest induced level was found under drought conditions, indicating CaDHN2 functions mainly in the drought response. Additionally, it was found that *CaDHN2* expression was also induced with ABA treatment (Figure 2C,D).

### 2.3. CaDHN2 Is Localized in the Plasma Membrane, Nucleus, and Cytoplasm 

**Figure 3:**
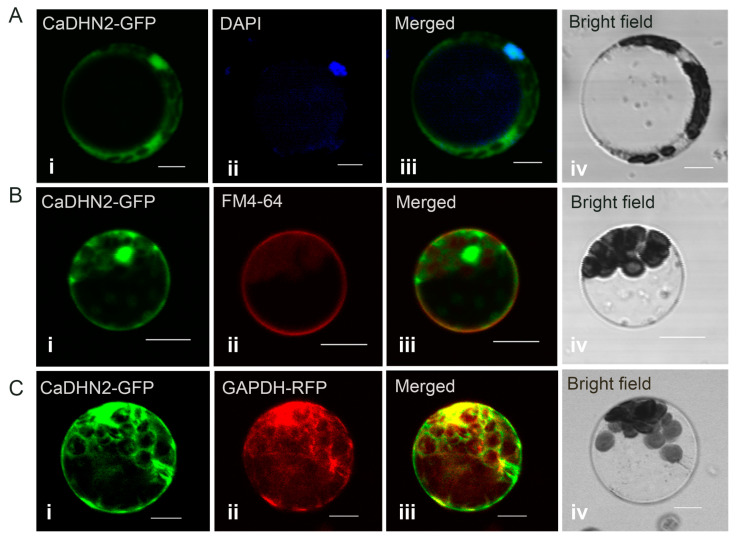
Subcellular localization of CaDHN2. (**A**) An *Arabidopsis* protoplast containing CaDHN2-GFP was stained with DAPI. The fluorescence signals were detected using confocal laser scanning microscopy with 488 nm for the GFP signal (**i**) and 518 nm excitation for DAPI (**ii**). (**iii**) Merged signal. (**iv**) Bright field. (**B**) A protoplast containing CaDHN2-GFP was stained with FM4-64. The fluorescence signals were detected using confocal laser scanning microscopy with 488 nm for the GFP signal (**i**) and 546 nm excitation for FM4-64 (**ii**). (**iii**) Merged signal. (**iv**) Bright field. (**C**) CaDHN2-GFP and GAPDH-RFP were co-transformed in an *Arabidopsis* protoplast. The fluorescence signals were detected using confocal laser scanning microscopy with 488 nm for the GFP signal (**i**) and 546 nm excitation for GAPDH-RFP (**ii**). (**iii**) Merged signal. (**iv**) Bright field. DAPI, 4′,6′-diamidino-2-phenylindole, a nuclear dye. FM4-64, a cellular membrane-specific lipophilic dye. GaAPDH was used as a marker of the cytoplasm. Bar: 10 μm.

In dehydrins, the S-segment is known to play a role in regulating nuclear localization, while the K-segment is important for membrane binding. To determine the subcellular localization of CaDHN2, CaDHN2-GFP was transformed into *Arabidopsis* protoplasts, and the fluorescence signals were detected using confocal laser scanning microscopy. Our results show that the green fluorescence signals were co-localized with DAPI staining, which is the specific dye for the nucleus (Figure 3A). Moreover, the green fluorescence signals were also co-localized with FM4-64 staining (Figure 3B), which is the specific dye for the plasma membrane. Additionally, we co-transformed CaDHN2-GFP and GAPDH-RFP in *Arabidopsis* protoplasts and found the green fluorescence signals were also co-localized with the red signals (Figure 3C), indicating that CaDHN2 is localized in cytoplasm since the GAPDH protein is widely distributed in the cytoplasm. These results indicate that CaDHN2 is localized in the plasma membrane, nucleus, and cytoplasm.

### 2.4. CaDHN2 Enhances Drought Tolerance in Capsicum annuum L.

To evaluate the impact of drought stress on pepper plants, we conducted a virus-induced gene silencing (VIGS) assay targeting CaDHN2 in the “P70” pepper cultivar. After a 30-day post-inoculation period, we observed photo-bleaching in the plants injected with pTRV2-PDS. Subsequently, we measured the relative expression levels of CaDHN2 using qRT-PCR in both pTRV2:00 (control) and pTRV2:CaDHN2 (silenced plants). The results showed a significant 65.72% reduction in the expression of CaDHN2 in the silenced plants compared with the control plants (Figure S1). This demonstrates the effective silencing of the CaDHN2 gene in the pepper specimens (Figure S1). 

**Figure 4:**
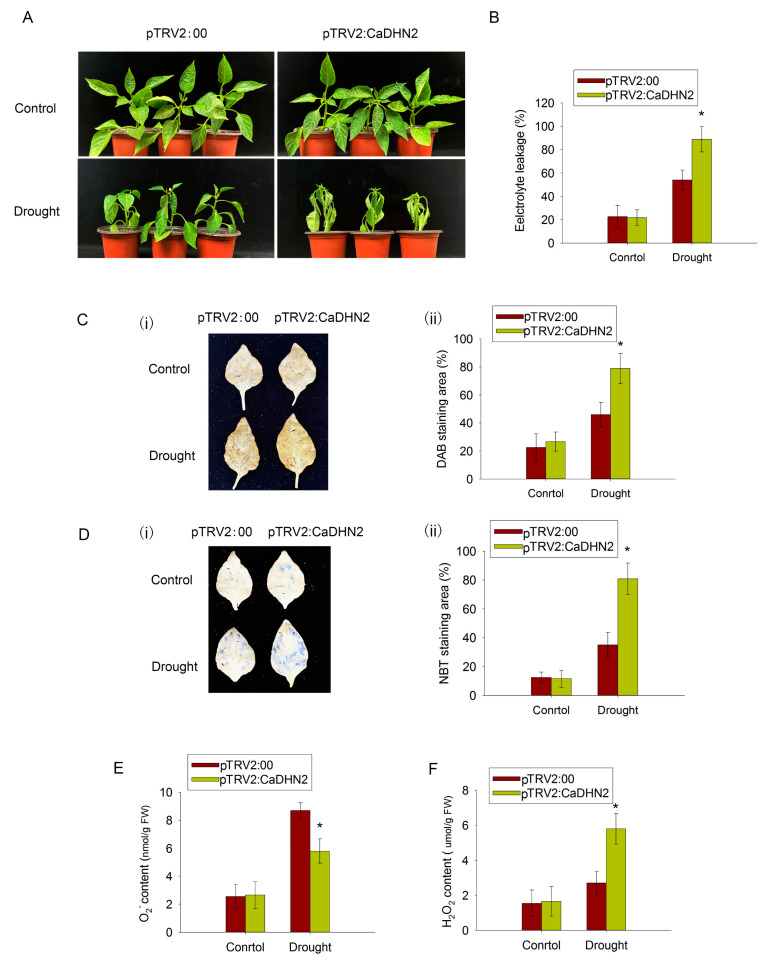
Silencing of CaDHN2 in pepper plants reduces drought tolerance and leads to increased accumulation of ROS. (**A**) Phenotypes of CaDHN2-silenced pepper plants (pTRV2:CaDHN2) and control plants (pTRV:00) under drought stress. (**B**) Electrolyte leakage rate of pTRV2:CaDHN2 plants and pTRV:00 plants. (**C**) DAB staining (**i**) and staining areas (**ii**). (**D**) NBT staining (**i**) and staining areas (**ii**). (**E**) O_2_^−^ contents of pTRV2:CaDHN2 plants and pTRV:00 plants under control and drought conditions. (**F**) H_2_O_2_ contents. Mean and SD values were obtained from three independent experiments. Asterisks indicate statistical significance compared with the control (* *p* < 0.05, Student’s *t*-test).

Under control conditions, no differences in phenotypic traits were observed between the pTRV2:00 and pTRV2:CaDHN2 plants. However, after 7 days of drought stress, the pTRV2:CaDHN2 pepper plants exhibited more wilting compared with the pTRV2:00 plants (Figure 4A). This suggests that the pTRV2:CaDHN2 pepper plants are more sensitive to drought stress.

The stability and integrity of cell membranes during water stress are crucial indicators of a plant’s ability to withstand drought, which is measured by relative electrolyte leakage. Under drought stress conditions, the pTRV2:CaDHN2 pepper plants exhibited significantly higher relative electrolyte leakage compared with the pTRV2:00 plants (Figure 4B). However, there were no differences in relative electrolyte leakage observed under control conditions (Figure 4B). These findings suggest that CaDHN2 promotes drought tolerance and acts as a positive regulator in the plant’s response to drought stress. 

Under stress conditions, reactive oxygen species (ROS) accumulate as part of the plant’s response. At low levels, ROS can function as signaling molecules involved in various physiological processes. However, when the ROS content reaches a higher level, it can cause damage to plants, leading to oxidative stress and causing the plants to go through ROS scavenging. DAB (3,3′-diaminobenzidine) and NBT (nitroblue tetrazolium) staining are commonly used methods to directly detect the accumulation of reactive oxygen species (ROS) in plant tissues. Our results indicated a significant increase in the stained areas for both DAB and NBT in the pTRV2:CaDHN2 pepper plants, ranging from approximately 34.99% to 56.20%, compared with the control (Figure 4C,D). This suggests that CaDHN2 plays a role in ROS scavenging. To confirm this result, the H_2_O_2_ and O_2_^−^ contents were measured in the pTRV2:CaDHN2 and pTRV2:00 plants. The findings demonstrated a significant increase in H_2_O_2_ levels within the pTRV2:CaDHN2 plants compared with the pTRV2:00 plants (Figure 4E). Conversely, the content of O_2_^−^ in the pTRV2:CaDHN2 plants was observed to be lower than that in the pTRV2:00 plants (Figure 4F).

Considering the important roles of superoxide dismutase (SOD), peroxidase (POD), and catalase (CAT) in the scavenging of reactive oxygen species (ROS), we measured the activity levels of these enzymes under drought-stress conditions. Our findings revealed significantly lower SOD, POD, and CAT activity in the CaDHN2-silenced pepper plants compared with the control plants. These results strongly suggest that CaDHN2 plays a crucial role in ROS scavenging during drought stress.

### 2.5. The Overexpression of CaDHN2 in Arabidopsis Enhances Drought Tolerance

**Figure 5:**
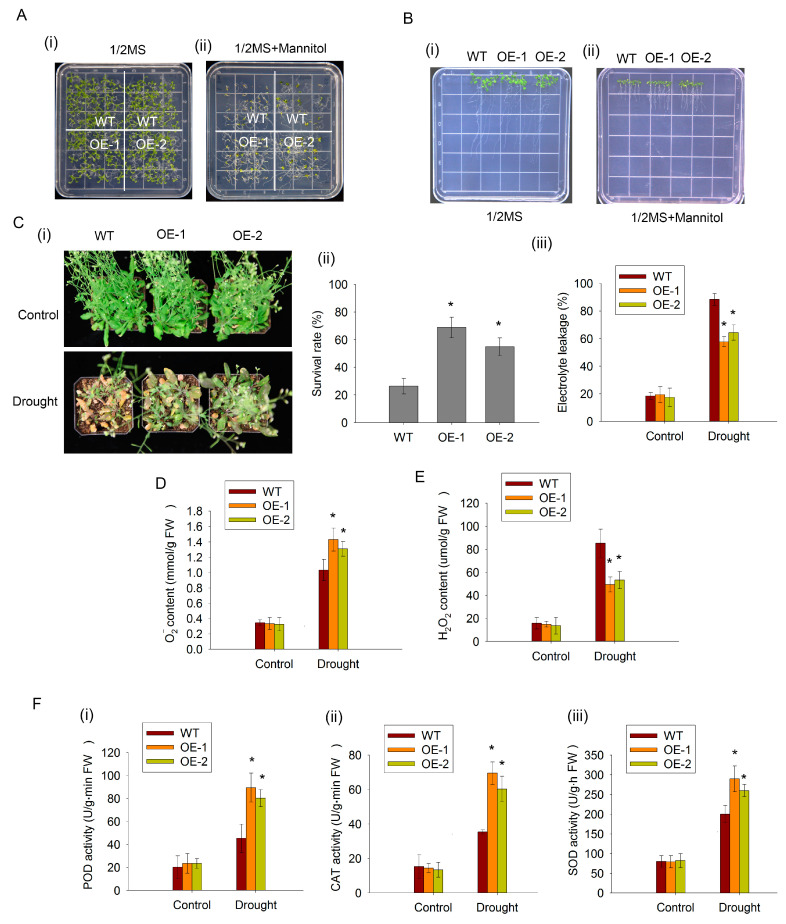
The overexpression of CaDHN2 in *Arabidopsis* enhanced the resistance to drought stress. (**A**) Germination of WT and OE in 1/2 MS (**i**) and 1/2 MS+mannitol (**ii**). (**B**) Root length of WT and OE in 1/2 MS and (**i**) 1/2 MS+mannitol (**ii**). (**C**) Phenotypes of WT and OE (**i**), survival rate (**ii**), and electrolyte leakage (**iii**) under control and drought conditions. (**D**) O_2_^−^ content. (**E**) H_2_O_2_ content. (**F**) Activity of antioxidant enzymes: (**i**) peroxidase (POD) activity, (**ii**) catalase (CAT) activity, and (**iii**) superoxide dismutase (SOD) activity. Mean and SD values were obtained from three independent experiments. Asterisks indicate statistical significance compared with the control (* *p* < 0.05, Student’s test).

To confirm the function of CaDHN2, we overexpressed CaDHN2 in Arabidopsis driven by a double CaMV35S promotor (OE). The CaDHN2-overexpressing lines and WT were subjected to drought stress. The germination rates of both OE plants and the WT plants were assessed on 1/2 MS solid medium containing 200 mM mannitol. We found that OE plants showed a significantly higher germination rate in response to the mannitol treatment (Figure 5A(ii)). However, under normal conditions (1/2 MS medium), no statistically significant differences in germination rates were observed (Figure 5A(i)). Moreover, the CaDHN2-overexpressing plants showed longer root lengths than the WT plants under mannitol treatments (Figure 5B). These results suggested that CaDHN2 plays a role in the drought response. To further confirm these findings, we conducted soil-grown experiments. After subjecting both the OE plants and the WT plants to drought treatment, we assessed the survival rates of the plants. Remarkably, the survival rate of the OE lines was significantly higher compared with the WT plants (Figure 5C(iii)). Consistent with the results, the CaDHN2-overexpressing lines exhibited higher electrolyte leakage compared with the WT plants under drought-stress conditions (Figure 5C(iii)). However, under normal conditions (Control), there was no difference observed (Figure 5C).

Since CaDHN2 plays a role in ROS scavenging in peppers (Figure 4), we also verified the results in CaDHN2-overexpressing *Arabidopsis*. Under normal growing conditions, there were no significant differences observed in ROS accumulation between OE and WT plants (Figure 5D,E). However, under drought stresses, the OE plants showed higher O_2_^−^ (Figure 5D) and lower H_2_O_2_ content than the WT plants (Figure 5E). Furthermore, under drought-stress conditions, significantly higher activity levels of POD (Figure 5F(i)), CAT (Figure 5F(ii)), and SOD (Figure 5F(iii)) were observed in the CaDHN2-overexpressing lines compared with the WT plants.

### 2.6. CaDHN2 Interacts with CaGGP1 Directly

**Figure 6:**
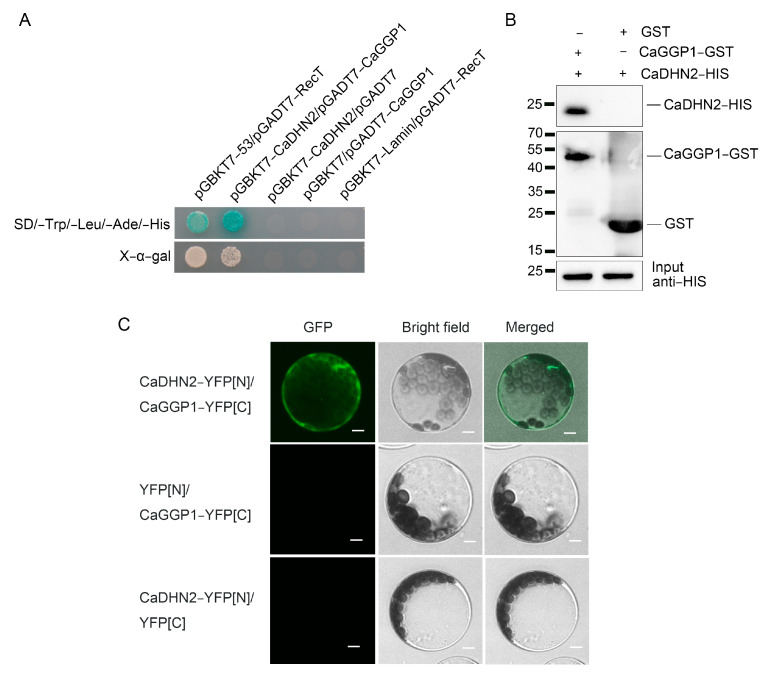
CaDHN2 interacts with CaGGP1. (**A**) Interaction between CaDHN2 and CaGGP1 in the yeast two-hybrid (Y2H) assay. (**B**) GST pull-down assay used to verify the interaction between CaDHN2 and CaGGP1. (**C**) Bimolecular fluorescence complementation (BIFC) assay to determine the interaction between CaDHN2 and CaGGP1. Bar: 10 μm.

In our previous studies, we attempted to improve the quality of pepper plants under drought-stress conditions. AsA, also known as vitamin C, is an essential component that contributes to the quality of peppers. Therefore, we specifically focused on evaluating the AsA content of peppers when subjected to drought stress. We have selected several proteins associated with ascorbic acid (AsA) synthesis to investigate the mechanism by which CaDHN2 enhances drought tolerance in *Capsicum annuum.* Fortunately, among the selected proteins, we discovered an enzyme, GGP1 (GDP-L-galactose phosphate 1), that plays a crucial role in the synthesis of ascorbic acid (AsA) and interacts with CaDHN2. Then, the interaction was verified using a yeast two-hybrid assay. We cloned CaDHN2 into the pGBKT7 vector (resulting in pGBKT7-CaDHN2) and CaGGP1 into the pGADT7 vector (creating pGADT7-CaGGP1). The co-transformed yeast strain AH109 was then cultured on selective media lacking tryptophan, leucine, histidine, and adenine (SD/-Trp/-Leu/-His/-Ade). The growth of the co-transformed yeast colonies on this selective medium indicated a positive interaction between CaDHN2 and CaGGP1 (Figure 6A). These results were also verified using a GST pull-down assay (Figure 6B) and a bimolecular fluorescence complementation (BiFC) assay (Figure 6C). 

### 2.7. CaDHN2 Protects CaGGP1 to Maintain AsA Synthesis under Drought Stress

Drought stress causes the degradation or aggregation of proteins, leading to metabolic and physiological disorders. AsA is a crucial antioxidant that plays a vital role in scavenging reactive oxygen species (ROS) and mitigating damage under stressful conditions.

**Figure 7:**
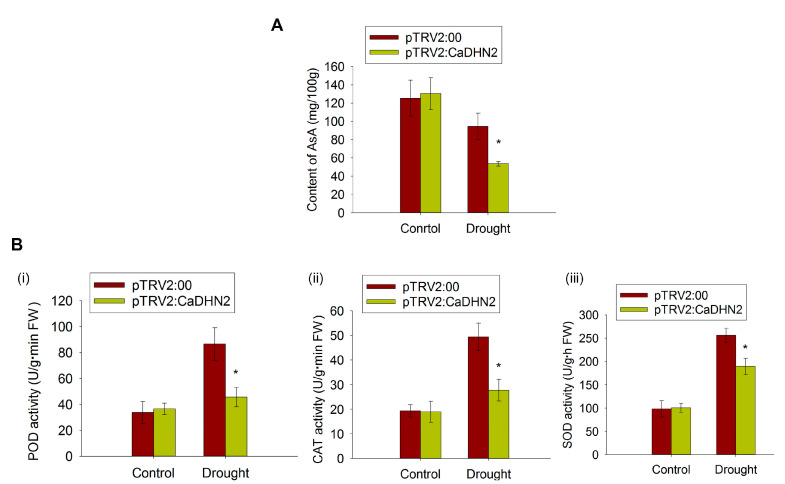
CaDHN2 maintains AsA synthesis under drought stress. (**A**) The content of AsA in CaDHN2-silenced pepper under normal conditions and drought stress at 40 DPA. (**B**) The activity of antioxidants: (**i**) POD activity, (**ii**) CAT activity, and (**iii**) SOD activity. Asterisks indicate statistical significance compared with the control (* *p* < 0.05, Student’s test).

As reported previously, dehydrins mainly function as cryo- and dehydration protectants, helping to maintain the normal metabolic activity in plants under stressful conditions [46]. So, we speculated that CaDHN2 may act as a protector of CaGGP1 through the interaction. Since CaGGP1 is the key enzyme in AsA synthesis, we measure the content of AsA in CaDHN2-silenced plants and control plants. The results clearly demonstrate that under drought stress at 40 DPA (days post-anthesis), the level of AsA is significantly higher in the control plants compared with the CaDHN2-silenced plants (Figure 7A). The increase in AsA content is associated with a decrease in ROS, as indicated by our results in Figure 4. We also measured the POD, CAT, and SOD activity in CaDHN2-silenced pepper plants and control plants. The results show that the activity of these antioxidant enzymes is much lower in CaDHN2-silenced pepper plants than in control plants under drought stress (Figure 7B). 

The chaperone or molecular shield function of dehydrin has already been demonstrated [47,48,49]. Dehydrins can prevent the aggregation or degradation of their interacting proteins under stress conditions [48,50], thereby maintaining normal physiological function. In our study, we discovered the chaperone activity of CaDHN2, which functions as a protein protector for CaGGP1. This chaperone activity helps maintain AsA synthesis, thereby enhancing the drought tolerance of pepper plants.

**Figure 8:**
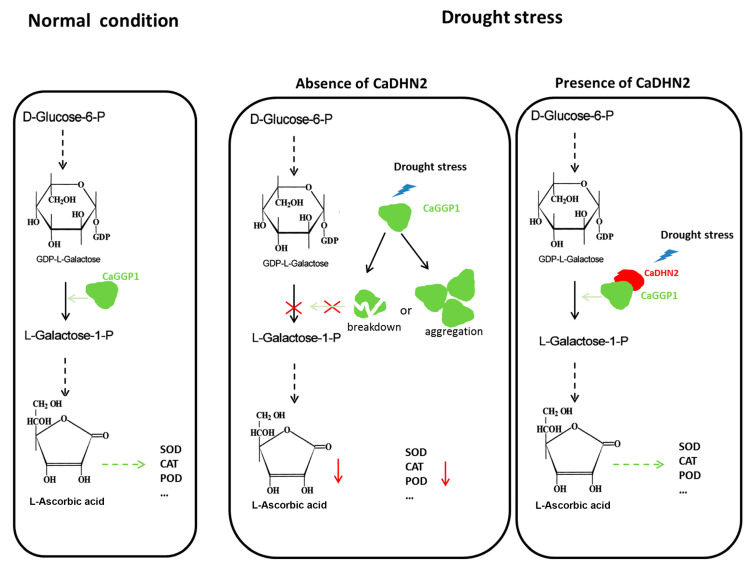
The function model of CaDHN2 in the pepper drought response under normal conditions. GDP-L-Galactose, synthesized from D-Glucose-6-P, undergoes conversion into L-Galactose-1-P, catalyzed by CaGGP1. This step is crucial for the subsequent synthesis of L-ascorbic acid (L-AsA). However, when pepper plants experience drought stress, CaGGP1 may undergo breakdown or aggregation in the absence of CaDHN2, thereby interrupting the AsA synthesis process. However, the presence of CaDHN2 plays a vital role as a protein protector, interacting with CaGGP1. This interaction effectively prevents the breakdown or aggregation of CaGGP1, ensuring the continuous synthesis of AsA. This protective mechanism helps maintain the normal physiological processes in pepper plants amidst drought-induced stress.

Taken together, we identified an SK_3_-type dehydrin, CaDHN2, which is up-regulated by drought stress and localized in the nucleus, cytoplasm, and plasma membrane. In pepper plants, CaDHN2 functions as a protein protector to interact with CaGGP1. The interaction between CaDHN2 and CaGGP1 may play a protective role, preventing the degradation or aggregation of CaGGP1 under drought-stress conditions. This interaction ensures the continuous synthesis of ascorbic acid under drought stress. The high levels of AsA increase the activity of CAT, SOD, and POD enzymes. This increased enzymatic activity assists pepper plants in scavenging ROS and enables them to withstand and survive under drought-stress conditions (Figure 8). 

## 3. Discussion

Dehydrin belongs to LEA group 2 and has been identified in numerous plant species, which have been subdivided into five categories: YnSKn, Kn, SKn, YnKn, and KnS. CaDHN2 contains one S-segment and three K-segments, classifying it as an SKn dehydrin. Different types of dehydrins exhibit variations in their subcellular or tissue localization. For instance, in wheat, the S-segment of dehydrins partially regulates the nuclear localization of WZY1-2 [51]. Additionally, K-segments play a vital role in membrane binding for dehydrins. When a K-segment was deprotonated of dehydrin Lti30, its ability to localize to the membrane was abolished [39]. In our studies, we observed that the SK_3_ dehydrin, CaDHN2 (Figure 1), is localized in the plasma membrane, cytoplasm, and nucleus (Figure 3). Furthermore, this type of dehydrin is also associated with different stress responses. YnSKn- and YnKn-type DHNs were found to be highly induced in response to ABA treatment. In wheat, YnSKn-type dehydrins, such as TaDHN1, TaDHN2, and TaDHN3, were observed to be significantly induced in response to NaCl (salt) treatment [52]. A Y2K4-type dehydrin, MtCAS31, exhibits up-regulation in response to various stress conditions including drought, salt, cold, and ABA treatment [40]. In our research, we discovered that the SK_3_-type dehydrin, CaDHN2, is not only induced by drought stress but also shows induction in response to ABA treatment (Figure 2).

Generally, dehydrins are characterized by their relatively high abundance of polar amino acids such as lysine, glycine, glutamine, serine, and proline. However, they are comparatively deficient in cysteine, isoleucine, leucine, and tryptophan. These compositional attributes contribute to the disordered conformation of dehydrins, making them highly hydrophilic and remarkably thermostable. The structural features are intimately related to their biochemical properties and functions. Dehydrins have been widely recognized for their crucial role in protecting cellular structures when plants are exposed to various abiotic stress factors. They display a diverse range of functions, including cryoprotection, antifreeze properties, radical scavenging, ion-binding capabilities, and the ability to act as both RNA and protein chaperones [50,53,54,55,56]. In *Arabidopsis*, dehydrins such as ERD10 and ERD14 function as chaperones to prevent the aggregation and inactivation of various proteins including lysozyme, alcohol dehydrogenase, firefly luciferase, and citrate synthase under stress conditions [50]. In our study, we observed that CaDHN2 interacts with CaGGP1 (Figure 6) and potentially acts as a chaperone. This chaperone function of CaDHN2 might help prevent the degradation or aggregation of CaGGP1, thereby maintaining the AsA content under drought stress conditions (Figure 7A).

The overexpression of dehydrins in various plant species, including crops like wheat and ornamental plants like Ammopiptanthus nanus, has been shown to enhance tolerance to drought, salt, and cold stresses. This indicates that the increased expression of dehydrins confers enhanced stress tolerance in different plant species across different contexts [42,57]. The silencing of CaDHNs has been observed to decrease the tolerance of plants to cold, salt, and drought stresses [17]. In our study, we found that *Arabidopsis* plants overexpressing CaDHN2 exhibited a higher survival rate under drought stress (Figure 5C(i,ii)). Additionally, these plants showed lower levels of electrolyte leakage (Figure 5C(iii)), indicating improved membrane integrity. In contrast, our study revealed that CaDHN2-silenced pepper plants (pTRV2:CaDHN2) exhibited increased sensitivity to drought stress compared with the control plants (Figure 5A,B). Various approaches have been used to investigate the protective role of dehydrins under stress conditions and enhance stress tolerance [37,58]. In the legume model plant *Medicago truncatula*, dehydrin MtCAS31 acts as a protective factor, preventing the degradation of leghemoglobin. This function helps to maintain the nitrogen fixation process under drought stress conditions [59]. In addition to their protective roles, new functions of dehydrins have also been identified. For instance, the dehydrin MtCAS31 can interact with the autophagy-related protein ATG8 and facilitate the process of autophagy degradation under drought-stress conditions [40]. This discovery provides us with a clue that dehydrins may play other vital roles in the response to abiotic stress beyond their known protective functions.

When plants are exposed to abiotic stressors, such as drought, salinity, or extreme temperatures, it often leads to an accumulation of ROS. These ROS molecules have the potential to cause oxidative damage to vital biomolecules within a plant’s cellular machinery, including lipids, proteins, and nucleic acids. So, the efficient scavenging of ROS is closely associated with a plant’s ability to withstand and exhibit tolerance toward abiotic stresses. To effectively scavenge ROS, plants respond by increasing the expression of antioxidant enzymes such as SOD, POD, and CAT. This upregulation occurs at both the transcriptional level and post-translationally. In our research, we observed that the silencing of CaDHN2 resulted in the accumulation of ROS under drought-stress conditions. The pTRV2:CaDHN2 pepper plants exhibited higher levels of H_2_O_2_ (Figure 4F) and lower levels of O_2_^−^ compared with the pTRV2:00 plants (Figure 4E). In contrast, the CaDHN2-overexpressing *Arabidopsis* lines displayed an opposite pattern, with reduced ROS accumulation under drought stress (Figure 5D,E). These findings suggest that CaDHN2 plays a crucial role in regulating ROS levels and oxidative stress response under drought conditions. 

AsA is involved in several crucial roles in plant physiology. Indeed, AsA serves as a conduit for transporting antioxidants and electrons within plants and also plays a critical role as an essential co-factor for various enzymes involved in plant metabolism [22,26,60]. Moreover, the application of AsA has been discovered to enhance the growth of plants and alleviate the detrimental effects caused by drought-induced stress conditions [23,61]. It possesses the capacity to enhance plant tolerance to abiotic stressors by promoting factors such as plant growth, the photosynthetic rate, the transpiration rate, oxidative defense mechanisms, and the maintenance of photosynthetic pigments. In our research, we provided evidence for the interaction between CaDHN2 and the key enzyme CaGGP1. This interaction is of particular significance because CaGGP1 is involved in the synthesis of AsA. The interaction implies a potential regulatory mechanism whereby CaDHN2 could have an impact on AsA biosynthesis. To support this notion, we measured the levels of AsA content and found that the presence of CaDHN2 correlated with changes in AsA levels under drought stress (Figure 7A). These findings suggest that CaDHN2 may play a role in modulating AsA biosynthesis, potentially contributing to the regulation of cellular AsA levels under drought conditions. AsA helps to maintain the active forms of SOD, POD, and CAT, enhancing their ability to scavenge and neutralize reactive oxygen species [29]. So, we measured the activities of antioxidant enzymes such as SOD, POD, and CAT under drought stress. Our results revealed that the pTRV2:CaDHN2 pepper plants exhibited lower activities of SOD, POD, and CAT compared with the pTRV2:00 plants under drought stress (Figure 7B). This suggests that the silencing of CaDHN2 in pepper plants might have a negative impact on the activation or functioning of these antioxidant enzymes, impairing their ability to scavenge ROS and attenuate oxidative stress induced by drought.

## 4. Material and Methods

### 4.1. Plant Materials

The *Capsicum annuum* L. cultivar “P70” was used in this research. To generate pTRV2:CaDHN2, a virus-induced gene silencing (VIGS) technique was used following a previously documented procedure [62,63]. In brief, a 320 bp segment from the 3′-untranslated region of CaDHN2 was inserted into a pTRV2 vector to construct the recombinant plasmid pTRV2:CaDHN2. Subsequently, an *Agrobacterium tumefaciens strain* (GV3101) was transformed with these plasmids, namely, pTRV2:00 (negative control) and pTRV2:CaDHN2, using the freeze–thaw method. The vectors above (pTRV2:00 and pTRV2:CaDHN2) were mixed with *A. tumefaciens*, which carries pTRV1. Pepper cotyledons were infiltrated with *Agrobacterium* suspensions (OD600 = 1.0) using a sterilized needleless syringe. The injected plants were initially kept in complete darkness at 18 °C for 2 days and then transferred to normal growth conditions (22 °C during the day for 16 h and 18 °C at night for 8 h) at 50% relative humidity. After approximately 30 days, both control pepper plants (pTRV2:00) and silenced plants (pTRV2:CaDHN2) were analyzed to determine the efficiency of gene silencing.

The silencing efficiency of VIGS is typically not sustained for an extended period and may only last for one or two months. In our research, we aimed to measure the ascorbic acid (AsA) content in pepper fruits of pTRV2:00 and pTRV2:CaDHN2 plants. To ensure a prolonged silencing period until the peppers reached their fruiting stage, VIGS was conducted twice on each pepper plant.

### 4.2. qRT-PCR

The relative expression of CaDHN2 was analyzed with quantitative real-time PCR (qRT-PCR) using a Bio-Rad CFX-96 real-time system and SYBR Premix ExTaq. Total RNA was extracted using TRIzol reagent, and cDNA was synthesized with reverse transcription using M-MLV reverse transcriptase. The expression levels of CaDHN2 were calculated using the 2^−ΔΔCt^ method and normalized to ACTIN gene expression. 

### 4.3. Stress Treatment

To investigate the function of CaDHN2, CaDHN2-silenced pepper plants (pTRV2:CaDHN2) and control plants (pTRV2:00) were subjected to drought stress. The plants were grown under a light/dark cycle of 16 h/8 h at temperatures of 22 °C during the day and 18 °C at night without water supply for 14 days and then were re-watered with 200 mL. Following this, another 14-day period of water withholding and re-watering was applied. After the drought treatment, several assays such as the electrolyte leakage, DAB staining, and NBT staining assays were conducted. 

To analyze the drought tolerance of CaDHN2-overexpressing *Arabidopsis*, we selected two transgenic lines (OE) and the WT plants. In the germination experiment, the WT and OE lines were grown on an MS medium containing 250 mM mannitol. To measure root length, the germinated WT and OE lines were transferred to the 1/2 MS medium containing 200 mM mannitol, and root length was determined on day 7. In the soil-based experiment, WT and OE seedlings were exposed to drought stress by withholding water for 14 days, followed by re-watering. Electrolyte leakage and the survival rate were recorded as indicators of tolerance to drought stress. The plants were grown under a 16-h light/8-h dark cycle at temperatures of 22 °C during the day and 18 °C at night. 

To investigate the expression pattern of CaDHN2 under stress conditions, we treated the pepper cultivar “P70” with mannitol, NaCl, and ABA to simulate various environmental stresses.

For the mannitol treatment, the roots of 4-week-old seedlings were immersed in 250 mM mannitol for 0.5 h, 1 h, 2 h, 4 h, 8 h, 12 h, 36 h, and 48 h, respectively. NaCl treatment was similar to the mannitol treatment: the roots of 4-week-old seedlings were soaked in 150 mM NaCl for 1 h, 6 h, 12 h, 24 h, and 48 h, respectively. For the ABA treatment, the leaves of the pepper plants were sprayed with a 10 μM ABA solution, and samples were collected at different time points following the treatment: 1 h, 6 h, 12 h, 24 h, and 48 h, respectively. In these treatments, the initial time point (0 h) represented untreated peppers, and the expression level of CaDHN2 at 0 h was used as the reference value (normalized as 1) was extracted from each sample and subjected to reverse transcription. The resulting cDNA was analyzed using quantitative real-time PCR (qRT-PCR) to assess the expression of CaDHN2.

### 4.4. Electrolyte Leakage

Electrolyte leakage was quantified following previously established procedures [64] using an ion leakage meter (HI8733, Hanna Rome, Italy,). Distilled water (10 mL) was added to a 50 mL centrifuge tube, and the electrolyte conductivity of the water was measured using an ion leakage meter. This initial measurement was marked as S0. Leaves detached from drought-treated and control plants were placed in a centrifuge. After suspending the leaves in the tubes for 2 h at room temperature, the electrolyte conductivity (S1) was measured. The centrifuge tubes were then heated in boiling water for 30 min. After cooling down, the electrolyte conductivity (S2) was measured. Relative electrolyte leakage was calculated as (S1 − S0)/(S2 − S0) × 100%.

### 4.5. Enzyme Activity

SOD and POD activities were assessed following a previously documented protocol [65,66]. Fresh leaves (0.5 g) were homogenized in 10 mL of PBS (pH 7.8), followed by centrifugation at 10,000 rpm for 15 min. The resulting supernatant was designated as the crude enzyme extract. For the assessment of POD activity, the extract was subjected to a spectrophotometric assay in the presence of hydrogen peroxide, which oxidizes guaiacol to generate colored products. The concentration of these products was measured to quantify POD activity. Similarly, SOD activity was determined utilizing a comparable approach, with NBT as the substrate. CAT activities were determined using a previously established methodology [67].

### 4.6. NBT and DAB Staining

NBT (nitro-blue tetrazolium) and DAB (3,3′-diaminobenzidine) staining were carried out to detect H_2_O_2_ and superoxide under drought stress. After staining, the leaves were de-stained by soaking them in 75% ethanol. After bleaching, images were taken as described before [68]. The quantification of the stained areas, using DAB and NBT, was performed following the methodology outlined by Sekulska-Nalewajko et al. [69]. 

### 4.7. Measurement of H_2_O_2_ and O_2_^−^

To measure the H_2_O_2_ and O_2_^−^ content, we followed the protocol described by Esfandiari et al. [70]. In brief, the samples were homogenized in 0.1% TCA (*w*/*v*). After homogenization, the resulting mixture was centrifuged at 12,000× *g* for 15 min, and then 0.5 mL of the supernatant was combined with 2 mL of 1 M KI and 0.5 mL of 100 mM potassium sulfate buffer. This mixture was kept in the dark for 1 h, and the absorbance was measured at 390 nm to quantify the H_2_O_2_ content. The quantification of O_2_^−^ content was conducted following the method outlined by Ke et al. [71].

### 4.8. Biomolecular Fluorescence Complementation (BiFC)

To investigate the interaction between CaGGP1 and CaDHN2 in vivo, biomolecular fluorescence complementation (BiFC) was utilized. CaGGP1 was inserted into the pSYCE vector, which fused with the N-terminus of GFP, resulting in pSYCE-CaGGP1. Similarly, CaDHN2 was inserted into the pSYNE vector, which was fused with the C-terminus of GFP, resulting in pSYNE-CaDHN2. The constructs were co-transfected into *Arabidopsis* protoplasts using PEG-mediated transformation. Following a 16-h incubation period, fluorescence signals were visualized with confocal laser scanning microscopy.

### 4.9. GST Pull-Down

In the protein pull-down experiments, the recombinant proteins (CaDHN2-HIS and CaGGP1-GST) were first purified from *Escherichia coli* (*E. coli*). The CaGGP1-GST protein was immobilized on glutathione beads and then incubated with CaDHN2-HIS in a binding buffer (comprising 20 mM Tris-HCl, pH 7.5, 3 mM MgCl_2_, 150 mM NaCl, 1 mM DTT, and 0.1% Triton X-100) [72] at 4 °C for 3 h. After incubation, the beads were washed three times using the binding buffer to remove any non-specifically bound proteins. Following the washing step, the samples were boiled and separated using SDS-PAGE. Immunoblotting techniques were then used to analyze the separated proteins using anti-HIS and anti-GST antibodies.

### 4.10. Yeast Two-Hybrid Assay

To ascertain the interaction between CaDHN2 and CaGGP1, a yeast two-hybrid assay was used. CaDHN2 was integrated into the pGBKT7 vector, and fused with the binding domain, resulting in pGBKT7-CaDHN2. CaGGP1 was inserted into the pGADT7 vector and fused with the activation domain, resulting in pGADT7-CaGGP1. These vectors were co-transformed into the AH109 strain using PEG-mediated transformation. The transformed yeast cells were plated on an SD medium lacking tryptophan, leucine, adenine, and histidine (SD/-Trp/-Leu/-Ade/-His). The yeast cultures were incubated at 30 °C for 48 h. Then, 20 mg/mL X-α-gal was used to assess the expression of the LacZ reporter gene.

### 4.11. Ascorbic Acid Content Measurement

For the quantification of ascorbic acid (AsA) concentration, high-performance liquid chromatography (HPLC) was used. AsA was extracted using metaphosphoric acid. Specifically, 0.5 g of pepper was utilized to prepare a 50 mL AsA solution in 0.1% metaphosphoric acid. This solution was then loaded onto a Reverse C18 column (250 mm × 4.6 mm, I.D. 5 μm) and eluted at a flow rate of 1 mL/min at 25 °C. The mobile phase consisted of a 0.02 mol/L phosphate buffer solution. AsA concentration was determined by measuring absorbance at 260 nm. AsA concentrations were quantified using a standard curve and expressed as milligrams per 100 g of fresh weight (FW). All samples were analyzed three times, and all procedures were conducted under dark conditions to prevent AsA oxidation.

### 4.12. Statistical Analysis

Statistical analysis of the data was performed using a *t*-test. The mean values ± standard deviation (SD) were calculated based on three independent biological replicates. Statistically significant differences compared with the control group are indicated as * *p* < 0.05.

## 5. Conclusions

In conclusion, our findings reveal the significant roles of CaDHN2 in ROS scavenging through the protection of AsA synthesis via its interaction with CaGGP1. Silencing CaDHN2 adversely affects AsA synthesis and subsequently reduces the activities of SOD, POD, and CAT enzymes, leading to increased ROS accumulation in plants. Consequently, a reduced tolerance of plants to drought stress is observed. This research provides valuable insights into the function of dehydrin and enriches our understanding of its role in controlling ascorbic acid content in pepper plants. Furthermore, it serves as a foundation for further studies exploring the involvement of dehydrins in responding to drought stress and offers meaningful clues regarding adaptability to various environmental stresses.

## Data Availability

Data are contained within the article and Supplementary Materials.

## Supplementary Materials

The following supporting information can be downloaded at: https://www.mdpi.com/article/10.3390/plants12223895/s1, Figure S1: The relative expression of CaDHN2 in pTRV2:CaDHN2. Statistical significance was assessed using Student’s *t*-test, comparing the obtained mean and standard deviation values from three independent experiments to the control. Significance is denoted with asterisks (* *p* < 0.05) in the results.
