# Supplementary material for: Dehydrin CaDHN2 Enhances Drought Tolerance by Affecting Ascorbic Acid Synthesis under Drought in Peppers
Source: Plants (Basel). 2023 Nov 18;12(22):3895. doi: 10.3390/plants12223895 (PMC10675185; doi:10.3390/plants12223895)
Supplement: Supplementary file 1 [file plants-12-03895-s001.zip › plants-2684795-supplementary.pdf]

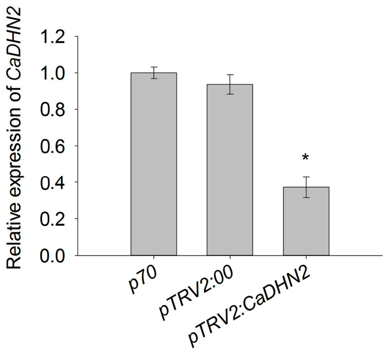


**Figure S1.** The relative expression of CaDHN2 in pTRV2:CaDHN2. Statistical significance was assessed using Student's *t*-test, comparing the obtained mean and standard deviation values from three independent experiments to the control. Significance is denoted with asterisks (* $p < 0.05$) in the results.
